# Electronegative LDL-mediated cardiac electrical remodeling in a rat model of chronic kidney disease

**DOI:** 10.1038/srep40676

**Published:** 2017-01-17

**Authors:** An-Sheng Lee, Wei-Yu Chen, Hua-Chen Chan, Ching-Hu Chung, Hsien-Yu Peng, Chia-Ming Chang, Ming-Jai Su, Chu-Huang Chen, Kuan-Cheng Chang

**Affiliations:** 1 Department of Medicine, Mackay Medical College, New Taipei, Taiwan; 2 Cardiovascular Research Laboratory, China Medical University Hospital, Taichung, Taiwan; 3 Graduate Institute of Biomedical Sciences, China Medical University, Taichung, Taiwan; 4 Center for Lipid Biosciences, Kaohsiung Medical University Hospital, Kaohsiung, Taiwan; 5 Graduate Institute of Pharmacology, National Taiwan University, Taipei, Taiwan; 6 Vascular and Medicinal Research, Texas Heart Institute, Houston, Texas, USA; 7 Lipid Science and Aging Research Center, Kaohsiung Medical University, Kaohsiung, Taiwan; 8 Center for Lipid Biosciences, Kaohsiung Medical University Hospital, Kaohsiung, Taiwan; 9 Division of Cardiovascular Medicine, Department of Medicine, China Medical University Hospital, Taichung, Taiwan; 10 Graduate Institute of Clinical Medical Science, China Medical University, Taichung, Taiwan

## Abstract

The mechanisms underlying chronic kidney disease (CKD)–associated higher risks for life-threatening ventricular tachyarrhythmias remain poorly understood. In rats subjected to unilateral nephrectomy (UNx), we examined cardiac electrophysiological remodeling and relevant mechanisms predisposing to ventricular arrhythmias. Adult male Sprague-Dawley rats underwent UNx (n = 6) or sham (n = 6) operations. Eight weeks later, the UNx group had higher serum blood urea nitrogen and creatinine levels and a longer electrocardiographic QTc interval than did the sham group. Patch-clamp studies revealed epicardial (EPI)-predominant prolongation of the action potential duration (APD) at 50% and 90% repolarization in UNx EPI cardiomyocytes compared to sham EPI cardiomyocytes. A significant reduction of the transient outward potassium current (*I*_to_) in EPI but not in endocardial (ENDO) cardiomyocytes of UNx rats led to a decreased transmural gradient of *I*_to_. The reduction of *I*_to_ currents in UNx EPI cardiomyocytes was secondary to downregulation of KChIP2 but not Kv4.2, Kv4.3, and Kv1.4 protein expression. Incubation of plasma electronegative low-density lipoprotein (LDL) from UNx rats with normal EPI and ENDO cardiomyocytes recapitulated the electrophysiological phenotype of UNx rats. In conclusion, CKD disrupts the physiological transmural gradient of *I*_to_ via downregulation of KChIP2 proteins in the EPI region, which may promote susceptibility to ventricular tachyarrhythmias. Electronegative LDL may underlie downregulation of KChIP2 in CKD.

Annual cardiovascular mortality in patients with chronic kidney disease (CKD) is much higher than that in the general population^1^,^2^. Sudden cardiac death (SCD) is the leading cause of cardiovascular mortality and may be responsible for 60% of cardiac deaths in patients undergoing dialysis^3^,^4^. The most common electrical mechanism leading to SCD involves the interaction of a triggering event and an abnormal cardiac substrate that induces ventricular tachycardia (VT), which degenerates into ventricular fibrillation (VF)^5^,^6^. Alternatively, SCD can be initiated directly by VF or polymorphic VT. SCD associated with bradyarrhythmias or asystole, often presenting as electromechanical dissociation, is less frequent and usually occurs in the setting of advanced heart failure^5^,^6^.

Even mild changes in renal function increase the risk of SCD, especially in older populations^7^; however, the mechanisms underlying this increased risk are unclear but may be attributed to the complications of atherosclerosis^8^. However, not all SCD cases are directly related to vascular dysfunction and myocardial infarction, suggesting the presence of other causes contributing to SCD^9^. Recently, Hsueh *et al*. used optical mapping to demonstrate the occurrence of abnormalities of cardiac electrical remodeling in a rat model of CKD^10^. The remodeling consisted of the loss of repolarization reserve and the presence of intracellular calcium abnormalities, which together may predispose to arrhythmia and SCD. However, the underlying mechanism is still unclear.

CKD may lead to dysregulation of key enzymes, transfer proteins, and receptors involved in lipoprotein metabolism; these changes cause multiple lipoprotein abnormalities^11^, which may occur during the early stages of CKD^12^,^13^. Dyslipidemia in CKD, characterized by high triglyceride and low high-density lipoprotein (HDL) levels and accumulation of small dense low-density lipoprotein (LDL) particles, is a shared risk factor for the development and progression of both CKD and cardiovascular diseases^14^. Our previous study showed that increased LDL electronegativity in CKD disrupts calcium homeostasis, resulting in cardiac diastolic dysfunction^15^. Furthermore, it has been reported that incubation of guinea pig ventricular myocytes with oxidized-LDL led to changes in electrophysiological properties including prolongation of the action potential duration (APD), depolarization of resting membrane potential, and modification of transmembrane ion currents^16^. Thus, in the present study, we hypothesize that electronegative LDL may play an important role in CKD-induced cardiac electrical remodeling.

Here, we have analyzed the electronegativity of plasma LDL and cardiac electrical remodeling in a rat model of early-stage CKD induced by unilateral nephrectomy (UNx) in both *in vitro* and *in vivo* experiments. To clarify the underlying electrophysiological mechanisms of CKD-related arrhythmia and SCD, we compared the characteristics of the transmural gradient of the APD and ion channels between cardiomyocytes isolated from the epicardial (EPI) and endocardial (ENDO) region by using the patch-clamp technique. The kinetic properties and protein expression levels of Kv1.4, Kv4.2, Kv4.3, and KChIP2 were also examined to investigate a possible influence of the repolarization K^+^ current in this animal model of CKD. Importantly, we compared the effects of LDL isolated from sham and UNx rats on freshly isolated rat cardiomyocytes to assess a causative role of electronegative LDL on CKD-related cardiac electrical remodeling.

## Results

### UNx rats exhibited prolonged QT intervals

**Figure 1:**
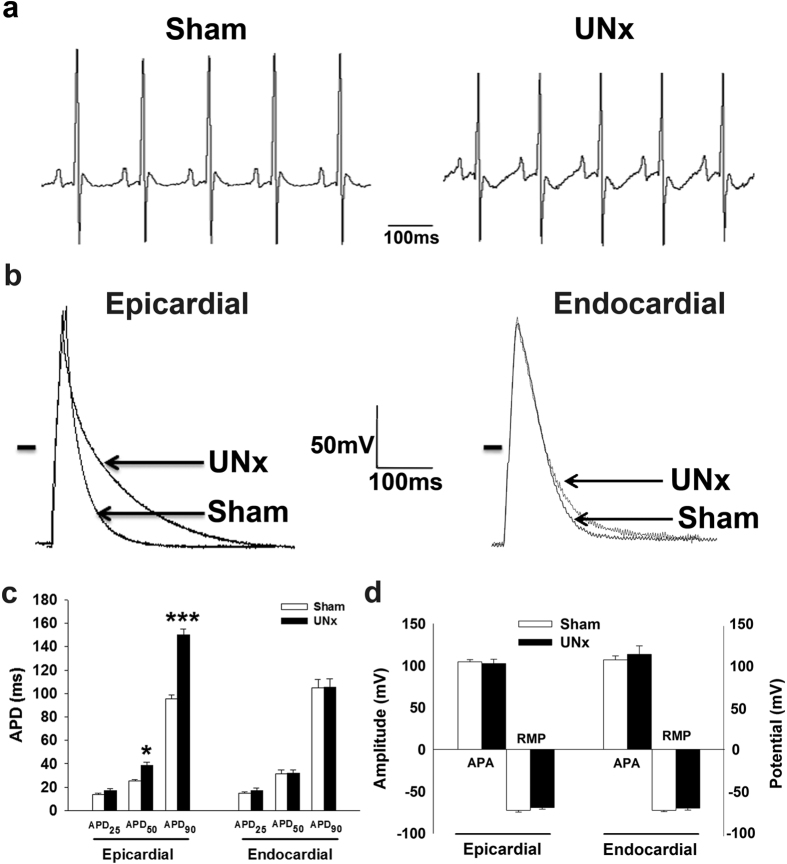
Prolongation of corrected QT interval and action potential duration in UNx rats. (**a**) Representative electrocardiogram recordings of sham and UNx rats. (**b**) Superimposed action potentials of epicardial (left panel) and endocardial (right panel) cardiomyocytes from sham and UNx rats. The horizontal line indicates zero voltage level. (**c**) Comparison of the action potential duration at 25% (APD_25_), 50% (APD_50_), and 90% (APD_90_) repolarization in epicardial and endocardial cardiomyocytes from both sham and UNx rats. (**d**) Comparison of the action potential amplitude (APA) and the resting membrane potential (RMP) in epicardial and endocardial cardiomyocytes from both sham and UNx rats. n = 10 from 6 animals per group. *P < 0.05, ***P < 0.01 vs. sham group.

We performed UNx on 6 adult Sprague-Dawley rats to create a rat model of early-stage CKD, and we performed a sham operation on 6 control rats. Eight weeks after the operation, body weight and total cholesterol, triglyceride, LDL, and HDL levels were not significantly different between UNx and sham rats (Table 1). However, blood urea nitrogen (BUN) and creatinine levels were significantly higher in UNx rats than in the sham group (*P* = 0.04 and *P* < 0.01, respectively). For surface electrocardiographic characteristics, the RR interval, heart rate, PR interval, P duration, and QRS interval were similar between UNx and sham rats (Fig. 1a). However, the QT interval and corrected QT interval (QTc) were prolonged by about 60% in UNx rats (0.082 ± 0.003 and 0.060 ± 0.002, respectively). We used Mitchell’s approach for the QTc analysis, which has been widely used at fast heart rates with consistent results in rodents^17^.

### Action potential duration was prolonged in EPI but not ENDO UNx cardiomyocytes

To further study the mechanisms involved in QT prolongation in UNx rats, we first compared action potentials between EPI and ENDO cardiomyocytes from sham and UNx rats by using the patch-clamp technique. Cardiomyocytes were paced with 3–5 ms suprathreshold depolarizing stimuli in the current-clamp mode. Figure 1b shows the superimposed recordings of action potentials for UNx and sham rat EPI and ENDO cardiomyocytes. The APD at 50% and 90% repolarization was longer in UNx EPI cardiomyocytes than in sham EPI cardiomyocytes (*P* = 0.048 and *P* = 0.008, respectively; Fig. 1c), but not significantly different between UNx and sham ENDO cardiomyocytes. No significant differences were observed between UNx and sham rat cardiomyocytes in action potential amplitude and resting membrane potential (Fig. 1d).

### Disruption of the transmural gradient of *I*_to_ by UNx

**Figure 2:**
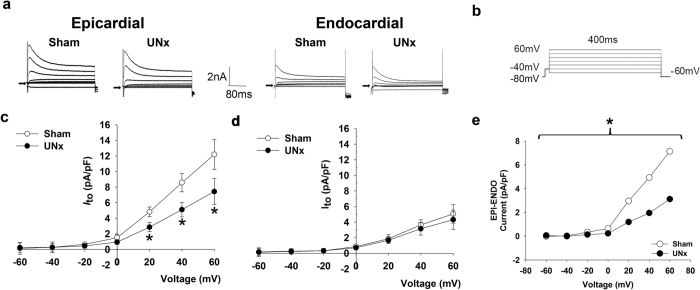
Altered transmural gradient of *I*_to_ in ventricular myocytes from UNx rats. (**a**) The original superimposed recordings of outward K^+^ current of epicardial (left panel) and endocardial (right panel) cardiomyocytes from sham and UNx rats. The arrow in each panel indicates zero current level. (**b**) Schematic diagram of the voltage clamp protocol. (**c**) Comparison of the I-V relationships of transient outward current (*I*_to_) in epicardial cardiomyocytes from sham and UNx rats. *P < 0.05 vs. sham group. (**d**) Comparison of the I-V relationships of transient outward current (*I*_to_) in endocardial cardiomyocytes from sham and UNx rats. n = 12 from 6 animals per group. (**e**) The difference between the mean epicardial (EPI) and endocardial (ENDO) *I*_to_ current of sham and UNx rats is plotted as the transmural gradient of *I*_to_. *P < 0.05 vs. sham rats, two-way ANOVA with the post-hoc Bonferroni correction.

The transient outward potassium current (*I*_to_) of cardiomyocytes isolated from ENDO and EPI regions of sham and UNx rats were recorded in the voltage-clamp mode (Fig. 2a). To determine *I*_to_, we elicited cardiomyocytes by a 10 ms step to −40 mV from the holding potential of −80 mV (to inactivate sodium channel), followed by a 400-ms depolarization to various potential levels ranging from −60 to 60 mV (applied at 20 mV increments every 1 s). The contamination of the calcium current was prevented by adding 1 mM Co^2+^ (Fig. 2b). Current-voltage relationships showed that *I*_to_ density was much larger in EPI than in ENDO cardiomyocytes in both sham and UNx rats, as predicted (Fig. 2c and d). However, EPI cardiomyocytes isolated from UNx showed a significantly smaller *I*_to_ at 60, 40, and 20 mV than those isolated from the corresponding layer in sham rats (*P* = 0.041, 0.033, and 0.045, respectively; Fig. 2c). In ENDO myocytes *I*_to_ density was similar in both groups (Fig. 2d). Figure 2e illustrates plots of the transmural gradient (EPI–ENDO) of *I*_to_ at different voltages from −60 to +60 mV for sham and UNx myocytes. The gradient was significantly smaller in cardiomyocytes from UNx than from sham animals (*P* = 0.021). We found no significant differences between sham and UNx cardiomyocytes in the *I*_Ca_ (−8.88 ± 1.02 vs. −9.57 ± 1.11 pA/pF at 10 mV, respectively; *P* = 0.649) and the *I*_K1_ currents (−38.16 ± 4.45 vs. −33.56 ± 2.51 pA/pF at −140 mV, respectively; *P* = 0.081) (Supplementary Fig. S1). In addition, sham LDL- and UNx LDL-treated normal cardiomyocytes had similar *I*_Ca_ (−18.99 ± 1.86 vs. −18.86 ± 3.41 pA/pF at 10 mV, respectively; *P* = 0.947) and *I*_K1_ currents (−32.11 ± 1.29 vs. −32.61 ± 2.43 pA/pF at −140 mV, respectively; *P* = 0.866) (Supplementary Fig. S2). Moreover, the midpoint of inactivation of *I*_Ca_ was similar between sham and UNx rats (−16.50 ± 1.90 vs. −16.23 ± 2.41; *P* = 0.933) and between sham LDL- and UNx LDL-treated cardiomyocytes (−16.00 ± 1.73 vs. −18.19 ± 2.76; *P* = 0.527) (Supplementary Fig. S3).

### Change in *I*_to_ gating properties in EPI cardiomyocytes from UNx rats

**Figure 3:**
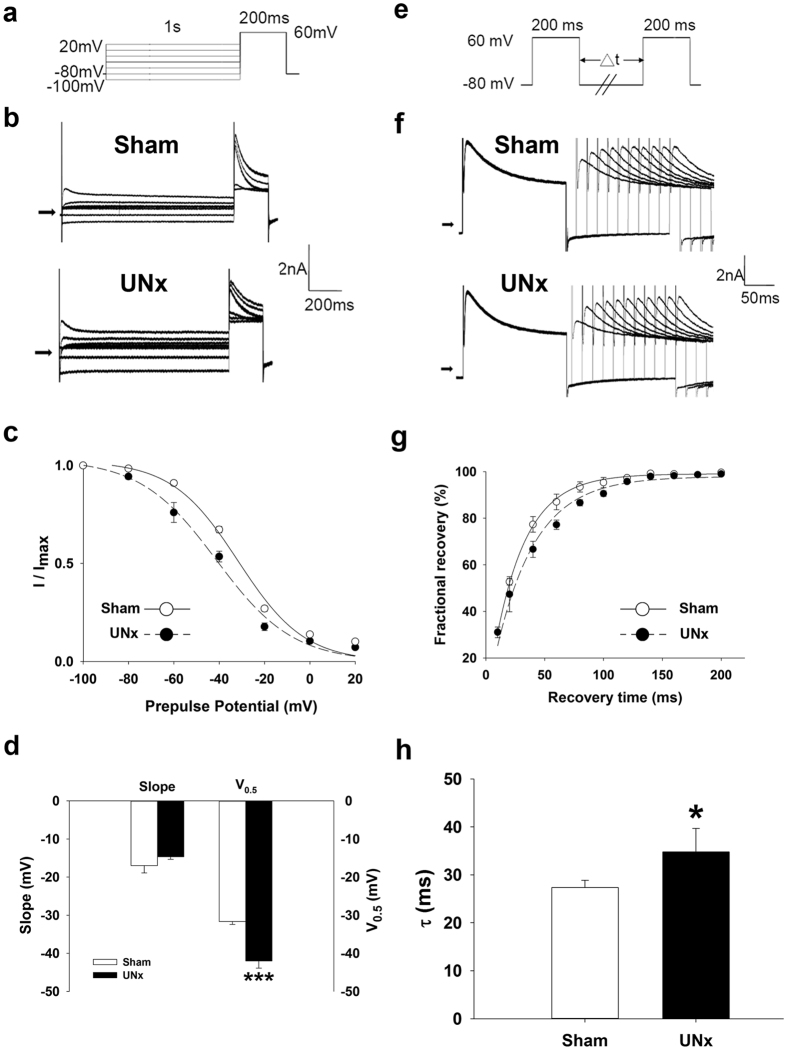
Kinetic change of *I*_to_ in epicardial cardiomyocytes from UNx rats. (**a**) Schematic diagram of the voltage clamp protocol of steady-state voltage-dependence inactivation. (**b**) The original superimposed recordings of steady-state inactivation trace in epicardial cardiomyocytes from sham and UNx rats. The arrow in each panel indicates zero current level. (**c**) The voltage-dependent steady-state inactivation curves for *I*_to_ were obtained by normalizing the current amplitudes to the maximal value and plotted as a function of the prepulse (conditioning) potentials. The lines drawn through the data points are the best fit to the Boltzmann equation. (**d**) Histogram comparing slope and midpoint voltage (V_0.5_) of each line between epicardial cardiomyocytes from sham and UNx rats. (**e**) Schematic of the typical 2-pulse protocol of kinetics of *I*_to_ recovery from inactivation. (**f**) The original superimposed recordings of recovery from inactivation in epicardial cardiomyocytes from sham and UNx rats. The arrow in each panel indicates zero current level. (**g**) The recovery from inactivation curves for *I*_to_ were obtained and fitted to a single exponential function. (**h**) Histogram comparing the average time constant for recovery from inactivation between epicardial cardiomyocytes from sham and UNx rats. n = 12 from 6 animals per group. *P < 0.05, ***P < 0.001 compared to sham group.

Because the smaller current density seen in EPI cardiomyocytes from UNx rats may be due to altered channel kinetics, we examined the gating properties of *I*_to_. To examine the voltage dependence of inactivation of *I*_to_, we used different voltages of conditioning pulses (−100 to +20 mV), which either depolarized or hyperpolarized from the holding potential of −80 mV for 1 second to inactivate the channel; with the second pulse, we depolarized to +60 mV for 200 ms from different precondition pulses (Fig. 3a). Figure 3b shows representative traces of *I*_to_ after different preconditioning voltages in EPI cardiomyocytes from sham and UNx rats. *I*_to_ amplitude normalized to the *I*_to_ of the most negative conditioning pulse (*I*_max_) was plotted against the conditioning voltage in Fig. 3c, and the curve was fitted by a Boltzmann model to obtain the midpoint of inactivation. We showed that the curve of UNx EPI cardiomyocytes was left-shifted and that the midpoint of inactivation was more negative than that of the sham group (*P* = 0.0004; Fig. 3d). For the kinetics of *I*_to_ recovery from inactivation, we used a typical 2-pulse protocol. Two identical pulses (from a holding potential of −80 mV to the test potential of +60 mV for 200 ms) were elicited in a variable interval from 0 to 200 ms in 20-ms increments (Fig. 3e). Representative traces of both sham and UNx EPI cardiomyocytes are provided in Fig. 3f, which showed *I*_to_ recovered from inactivation at a much slower rate in EPI cardiomyocytes from UNx rats than in those from the sham group. Normalized currents were plotted against the interval between the two pulses, and the data were fitted by a single exponential equation (Fig. 3g). The time constant (τ) of UNx EPI cardiomyocytes was significantly increased when compared to that in the sham group (*P* = 0.04; Fig. 3h). In ENDO cardiomyocytes, the inactivation and recovery curves of sham and UNx were comparable, and there were no significant differences between the two groups (data not shown).

### Incubation of LDL from UNx rats prolonged action potential in normal EPI but not ENDO cardiomyocytes

**Figure 4:**
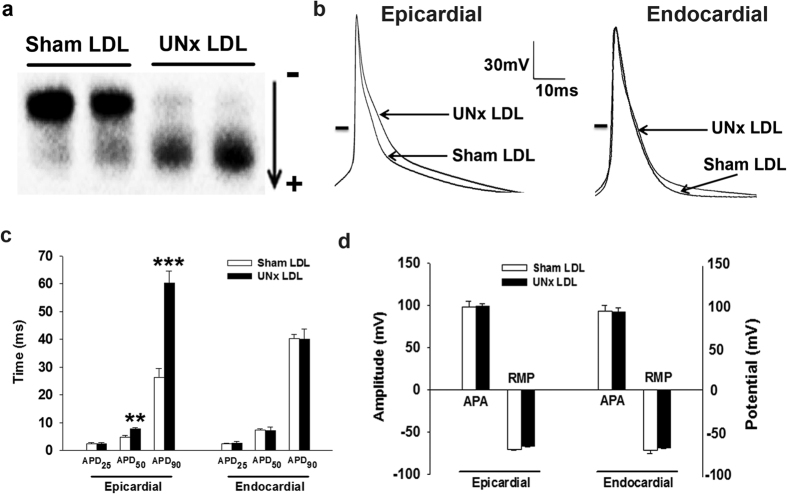
Electronegativity of LDL isolated from UNx rats and its effects on action potential. (**a**) Representative agarose gel electrophoresis of LDL isolated from UNx and sham rats. (**b**) Superimposed action potentials of normal epicardial (left panel) and endocardial (right panel) cardiomyocytes treated with 100 μg/mL sham LDL and UNx rat LDL. The horizontal line indicates zero voltage level. (**c**) Comparison of the action potential duration at 25% (APD_25_), 50% (APD_50_) and 90% (APD_90_) repolarization in epicardial and endocardial cardiomyocytes treated with sham LDL and UNx LDL. (**d**) Comparison of the action potential amplitude (APA) and resting membrane potential (RMP) in epicardial and endocardial cardiomyocytes treated with sham LDL and UNx LDL. n = 6 from 4 animals per group. **P < 0.01, ***P < 0.001 vs. sham LDL group.

To clarify the underlying mechanisms of UNx-induced electrophysiologic remodeling and to determine whether circulating LDL plays an important role, we compared the electronegativity of LDL between sham and UNx rats. The results of agarose gel electrophoresis showed that the LDL from UNx rats was more electronegative than that from healthy controls (Fig. 4a). We then incubated normal EPI cardiomyocytes with 100 μg/mL LDL from sham or UNx rats for 12 hours. Figure 4b shows the superimposed recordings of action potentials for UNx LDL- and sham LDL-treated normal EPI and ENDO cardiomyocytes. The APD at 50% and 90% repolarization was longer in UNx LDL-treated than in sham LDL-treated EPI cardiomyocytes (*P* = 0.002 and *P* = 0.0001, respectively; Fig. 4c) but was not significantly different between UNx LDL- and sham LDL-treated ENDO cardiomyocytes. No significant differences were observed in action potential amplitude and resting membrane potential between UNx LDL- and sham LDL-treated EPI and ENDO cardiomyocytes (Fig. 4d).

### Incubation of EPI myocytes with LDL from UNx but not sham rats reduced *I*_to_ density and its transmural gradient

**Figure 5:**
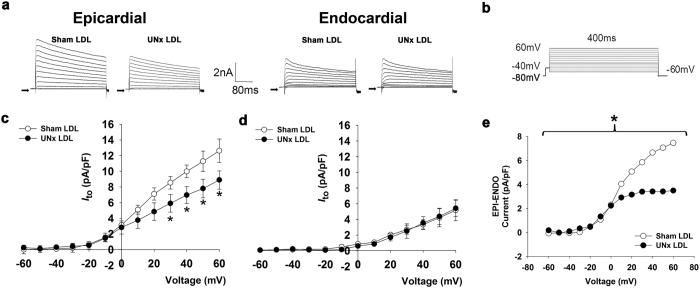
Disruption of transmural gradient of *I*_to_ in ventricular myocytes treated with UNx rat LDL. (**a**) The original superimposed recordings of outward K^+^ current of normal epicardial (left panel) and endocardial (right panel) cardiomyocytes treated with 100 μg/mL sham and UNx rat LDL. The arrow in each panel indicates zero current level. (**b**) Schematic diagram of the voltage clamp protocol. (**c**) Comparison of the I-V relationships of transient outward current (*I*_to_) in epicardial cardiomyocytes treated with sham and UNx rat LDL. *P < 0.05 vs. sham LDL group. (**d**) Comparison of the I-V relationships of transient outward current (*I*_to_) in endocardial cardiomyocytes treated with sham and UNx rat LDL. (**e**) The difference between the mean epicardial (EPI) and endocardial (ENDO) *I*_to_ current of sham and UNx rat LDL-treated cardiomyocytes is plotted as the transmural gradient of *I*_to_. n = 7 from 5 animals per group. *P < 0.05 vs sham LDL group, two-way ANOVA with the post-hoc Bonferroni correction.

The transient outward potassium current (*I*_to_) of both EPI and ENDO cardiomyocytes treated with sham LDL and UNx LDL was recorded (Fig. 5a). For the determination of *I*_to_, we elicited cardiomyocytes by a 10 ms step to −40 mV from the holding potential of −80 mV, followed by 400 ms depolarization to various potential levels ranging from −60 to 60 mV (applied at 10 mV increments every 1 s) (Fig. 5b). Current-voltage relationships showed that *I*_to_ density at 60, 50, 40, and 30 mV was significantly smaller in UNx LDL-treated EPI cardiomyocytes than in sham LDL-treated EPI cardiomyocytes (*P* = 0.020, 0.038, 0.038, and 0.045, respectively; Fig. 5c). In ENDO cardiomyocytes *I*_to_ density was similar in both groups (Fig. 5d). Figure 5e shows the plots of the transmural gradient of *I*_to_ at different voltages from −60 to +60 mV for sham LDL- and UNx LDL-treated cardiomyocytes. The gradient was significantly smaller in UNx LDL-treated cardiomyocytes than in sham LDL-treated cardiomyocytes (*P* = 0.029).

### Incubation of EPI cardiomyocytes with LDL from UNx but not sham rats alters *I*_to_ so that its gating properties resemble those found in UNx EPI cardiomyocytes

**Figure 6:**
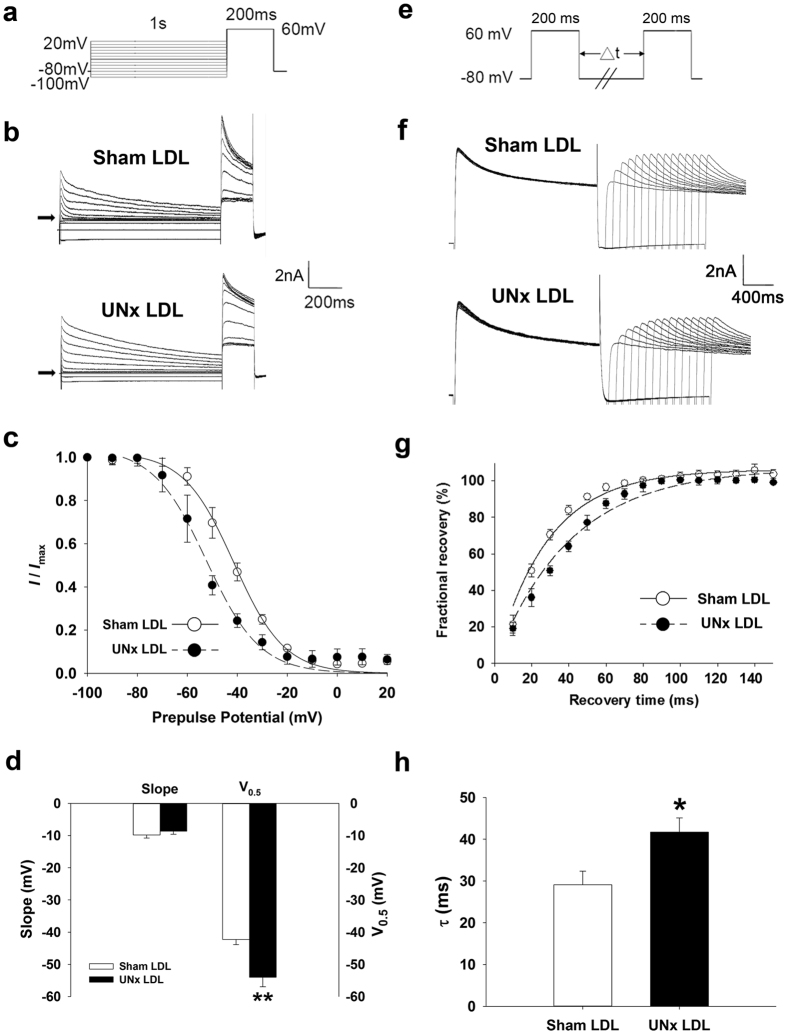
Kinetic change of *I*_to_ in normal epicardial cardiomyocytes treated with UNx rat LDL. (**a**) Schematic diagram of the voltage clamp protocol of steady-state voltage-dependence inactivation. (**b**) The original superimposed recordings of steady-state inactivation trace in epicardial cardiomyocytes treated with 100 μg/mL sham and UNx rat LDL. The arrow in each panel indicates zero current level. (**c**) The voltage-dependent steady-state inactivation curves for *I*_to_ were obtained by normalizing the current amplitudes to the maximal value and plotted as a function of the prepulse (conditioning) potentials. The lines drawn through the data points are the best fit to the Boltzmann equation. (**d**) Histogram comparing slope and midpoint voltage (V_0.5_) of each line between epicardial cardiomyocytes from sham and UNx rats. (**e**) Schematic of the typical 2-pulse protocol of kinetics of *I*_to_ recovery from inactivation. (**f**) The original superimposed recordings of recovery from inactivation in epicardial cardiomyocytes from sham and UNx rats. The arrow in each panel indicates the zero current level. (**g**) The recovery from inactivation curves for *I*_to_ were obtained and fitted to a single exponential function. (**h**) Histogram comparing the average time constant for recovery from inactivation between epicardial cardiomyocytes from sham and UNx rats. n = 7 from 5 animals per group. *P < 0.05, **P < 0.01 compared to sham LDL group.

We compared the gating properties of *I*_to_ between sham LDL- and UNx LDL-treated cardiomyocytes to clarify the mechanism underlying *I*_to_ downregulation. To examine the voltage dependence of inactivation of *I*_to_, we used different voltages of conditioning pulses (−100 to +20 mV), which either depolarized or hyperpolarized from the holding potential of −80 mV for 1 second to bring the membrane to inactivation; with the second pulse, we depolarized to +60 mV for 200 ms from different precondition pulses (Fig. 6a). Figure 6b shows representative traces of *I*_to_ after different preconditioning voltages in sham LDL- and UNx LDL-treated EPI cardiomyocytes. *I*_to_ amplitude normalized to the *I*_to_ of the most negative conditioning pulse (*I*_max_) was plotted against conditioning voltage in Fig. 6c, and the curve was fitted by a Boltzmann model to obtain the midpoint of inactivation. We showed that the curve of UNx LDL-treated EPI cardiomyocytes was left-shifted and that the midpoint of inactivation was more negative than that of the sham LDL group (*P* = 0.002; Fig. 6d). For the kinetics of *I*_to_ recovery from inactivation, we used a typical 2-pulse protocol. Two identical pulses (from holding potential of −80 mV to the test potential of +60 mV for 200 ms) were elicited in variable intervals from 0 to 140 ms in 10-ms increments (Fig. 6e). Representative traces of both sham LDL- and UNx LDL-treated EPI cardiomyocytes are presented in Fig. 6f, which shows *I*_to_ recovered from inactivation at a much slower rate in UNx LDL-treated EPI cardiomyocytes than in the sham LDL-treated group. Normalized currents were plotted against the interval between two pulses, and the data were fitted by a single exponential equation (Fig. 6g). The time constant (τ) of UNx LDL-treated EPI cardiomyocytes was significantly increased when compared to that of the sham LDL group (*P* = 0.02; Fig. 6h). Similar to those found in UNx ENDO cardiomyocytes, the inactivation and recovery curves of sham LDL- and UNx LDL-treated cardiomyocytes were comparable, and there were no significant differences between the groups (data not shown).

### KChIP2 downregulation in EPI layer of UNx rats and UNx LDL-treated EPI cardiomyocytes

**Figure 7:**
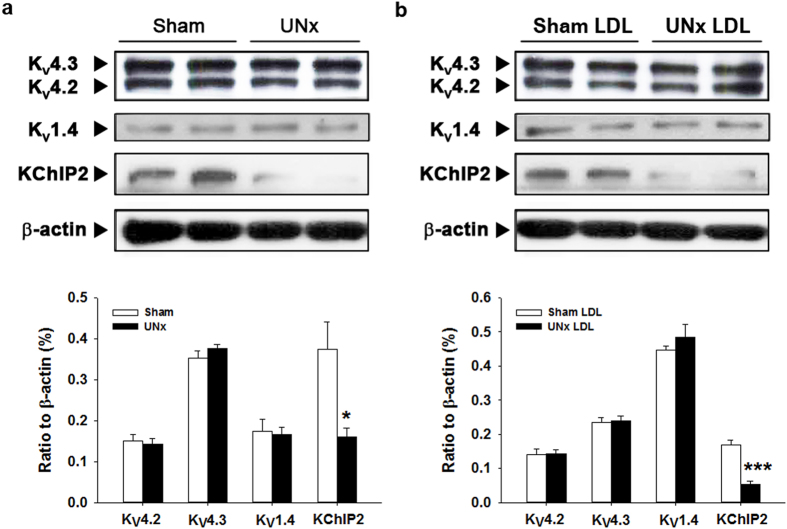
Effect of unilateral nephrectomy (UNx) on *I*_to_-related protein expression. (**a**) Representative western blot showing KV4.3, KV4.2, KV1.4, and KChIP2 expression and the quantitative analysis in epicardial cardiomyocytes from sham and UNx rat. *P < 0.05 vs. sham group. (**b**) Representative western blot showing KV4.3, KV4.2, KV1.4, and KChIP2 expression and the quantitative analysis in normal epicardial cardiomyocytes treated with 100 μg/mL sham and UNx rat LDL. n = 5 per group. ***P < 0.001 vs. sham LDL group. All western blot data were cropped and acquired under the same experimental conditions. The full-length blots are presented in the supplementary information (Supplementary Fig. S4).

To determine the mechanism underlying changes of *I*_to_ channel properties in the EPI layer of UNx rats and UNx LDL-treated EPI cardiomyocytes, we examined the expression levels of Kv4.3, Kv4.2, Kv1.4, and KChIP2 proteins in the EPI layer from sham or UNx rats (Fig. 7a) and sham LDL- or UNx LDL-treated EPI cardiomyocytes (Fig. 7b). In EPI cardiomyocytes from UNx rats, the expression of Kv4.3, Kv4.2, and Kv1.4 proteins was comparable with that in sham myocytes, suggesting that Kv α-subunits did not contribute to the downregulation of *I*_to_. However, the expression of β-subunits KChIP2 protein was significantly reduced in UNx EPI cardiomyocytes compared to sham cardiomyocytes (*P* = 0.017). Similarly, 100 μg/mL UNx LDL incubation could reduce KChIP2 protein expression (*P* = 0.0001) but did not change Kv4.3, Kv4.2, and Kv1.4 expression in control EPI cardiomyocytes. There were no significant differences in both α- and β-subunits of *I*_to_ between ENDO cardiomyocytes from sham and UNx rats or between sham LDL- and UNx LDL-treated ENDO cardiomyocytes (data not shown).

## Discussion

In rats with UNx-induced CKD, we have demonstrated electrocardiographic QTc prolongation *in vivo* that is mediated by a predominant lengthening of the APD in EPI cardiomyocytes. The differential reduction of *I*_to_ currents between EPI and ENDO cardiomyocytes in UNx rats, along with the resultant disruption of the physiological transmural gradient of *I*_to_, may contribute to the APD phenotype. The electrophysiological remodeling with substantial *I*_to_ reduction and alteration of transmural *I*_to_ gradient in epicardial cardiomyocytes was reproduced in UNx-LDL treated cardiomyocytes. The reduction of *I*_to_ is not due to altered expression levels of Kv4.3, Kv4.2, or Kv1.4 proteins, but rather it is related to downregulation of the Kv channels regulator KChIP2. Taken together, the predominant downregulation of KChIP2 protein and *I*_to_ currents in EPI cardiomyocytes led to alteration of the transmural *I*_to_ gradient, which may create a vulnerable substrate for generating life-threatening ventricular tachyarrhythmias in CKD.

### QTc prolongation in CKD

The progression of CKD has been associated with a significant delay of cardiac repolarization manifested by QTc prolongation, independent of other risk factors or structural heart diseases in humans^18^. The prolongation of QTc can occur even in patients with early-stage CKD (stage 2)^19^. The abnormalities in cardiac repolarization may thus predispose susceptible CKD patients to fatal ventricular tachyarrhythmias. In support of the human data, in a rat model of CKD with a defect in the samcystin gene (Cy/+), Hsueh *et al*.^10^ showed that the APD at 80% of repolarization was longer in CKD rats than in normal rats. Pacing cycle length thresholds to induce calcium transient alternans or APD alternans were also longer in CKD rats. This electrophysiological remodeling led to increased vulnerability to ventricular arrhythmia characterized by the spontaneous occurrence of premature ventricular complexes and a higher frequency of VF induction. Although this model of genetic defect–induced CKD has shown some remodeling abnormalities including altered repolarization reserve and calcium homeostasis by optical mapping technique, the underlying mechanism remains unclear.

In contrast to the study by Hsueh *et al*., we used a standard UNx-induced CKD model to examine CKD-related cardiac electrophysiological remodeling by the patch-clamp technique. In rodents, APD has been determined mostly by the density of the Ca^2+^ -independent *I*_to_ current^20^. However, *I*_to_ is not uniformly distributed among the whole ventricle. Rather, *I*_to_ is more prominent in the sub-epicardial region than in the sub-endocardial tissue in rodent hearts, which causes the baseline APD to be shorter in the EPI region than in the ENDO region^21^. During some pathological conditions, such as cardiac hypertrophy, the regional difference of *I*_to_ reduction can reverse the gradient in APD and ultimately contribute to the increased susceptibility to ventricular arrhythmias^22^,^23^. In this early-stage CKD model, we demonstrated that the overall electrocardiographic QTc was prolonged in CKD rats compared to sham-operated rats; this prolongation was mediated by an extensive lengthening of APD in EPI cardiomyocytes compared with ENDO cardiomyocytes and was secondary to a substantial reduction of *I*_to_ in the EPI region. The finding that modulation of *I*_to_ leads to prolongation or abbreviation of APD was concordant with the observation by Li *et al*.^24^, who showed that ablation of cold-inducible, RNA-binding protein–mediated *I*_to_ amplification shortened the QTc interval in rats. In the current study, the changes in electrophysiological properties in CKD rats occurred independently of ventricular fibrosis because fibrosis levels were similar in CKD and control rats, as shown by histologic staining and examination of early fibrosis markers^15^. This electrophysiological remodeling in CKD rats also occurred regardless of cellular hypertrophy based on the finding of similar cellular capacitance in isolated single ventricular cardiomyocytes by the patch-clamp technique (200.69 ± 9.62 in sham vs. 190.37 ± 12.07 pA/pF in UNx). Moreover, Yu *et al*. found that the renin-angiotensin-aldosterone system (RAAS) could alter the transmural gradient of *I*_to_ between EPI and ENDO cardiomyocytes by changing channel kinetics^25^. In our CKD model, the levels of renin, angiotensin, and aldosterone were not significantly different between UNx and sham rats^15^. This finding indicates that the electrophysiological remodeling seen in UNx rats was not mediated by modulation of the RAAS.

### KChIP2 downregulation disrupts physiological transmural gradient of *I*_to_ in CKD

There are species-specific differences in voltage-gated K^+^ channel expression that are responsible for the timely control of cardiac repolarization. Downregulation of these currents results in prolongation of the APD and a decrease in phase 1 repolarization^26^. Significant levels of mRNA encoding the rapidly inactivating K^+^ channel α-subunits Kv1.4, Kv4.2, and Kv4.3 proteins have been seen in rat ventricle^27^. In addition, previous studies have identified three classes of distinctive cytoplasmic auxiliary proteins, including KChIPs, Kv β-subunits, and KChAP. These proteins not only incorporate with specific Kv α-subunits to generate the native *I*_to_ current, but also regulate functional expression of these channels *in vitro*^28^,^29^,^30^.

Previous *in vitro* studies have examined the modulatory effects of the KChIP2 protein on the gating kinetics of Kv4 channels in heterologous expression systems. An *et al*.^28^ showed that KChIP proteins may co-localize with Kv4 a-subunits in transiently transfected COS-1 cells. The effects of KChIP1, 2 and 3 may augment A-type currents of Kv4 channels via a variety of mechanisms including increased surface channel density, shifting the activation V_1/2_ to more negative potentials, and slower inactivation and faster recovery from inactivation in native cells. Schultz *et al*.^31^ have recorded whole-cell currents from oocytes injected with porcine Kv4.2 cRNA alone or co-injected with porcine Kv4.2 and porcine KChIP2 cRNA. They found that steady-state inactivation was only marginally, not significantly, affected by KChIP2, whereas the recovery from inactivation was significantly accelerated by co-expressing Kv4.2 with KChIP2.

The reduction in *I*_to_ density with the change in channel kinetics in cardiomyocytes from UNx rats and UNx LDL-treated cardiomyocytes suggests that the molecular level of *I*_to_ is altered. Previous studies have shown that the differential expression of both KChIP2 and Kv4.2 proteins across the ventricular wall may determine the transmural *I*_to_ gradient in mouse, rat, canine, and human hearts^32^,^33^,^34^. In a rat model of type 2 diabetes, Sato *et al*.^34^ found an ENDO-predominant prolongation of the APD via a reduction of steady-state *I*_to_ induced by type 2 diabetes, in which downregulation of Kv4.2 and KChIP2 proteins may be involved. By using the rat model of early CKD, we discovered an EPI-predominant lengthening of the APD via a reduction of *I*_to_ currents in EPI cardiomyocytes secondary to downregulation of KChIP2 without alteration of Kv4.2, Kv4.3, and Kv1.4 protein expression. The reduction of *I*_to_ currents caused by KChIP2 deficiency may be attributed to shifting the voltage dependence of inactivation to more hyperpolarized potentials and slower recovery from inactivation as shown in the current and previous studies^28^,^29^. Importantly, downregulation of KChIP2 protein abolishes the transmural gradient of *I*_to_ leading to increased heterogeneity of repolarization; this may in turn cause unidirectional conduction block, creating a substrate for the reentry circuit and thus increasing susceptibility to malignant ventricular tachyarrhythmias^32^.

### Electronegative LDL may underlie downregulation of KChIP2 protein expression in CKD

It is generally thought that during the course of CKD, the kidney releases mediators that trigger maladaptive myocardial remodeling, ultimately leading to adverse cardiovascular outcomes. In addition to the well-known inflammatory mediators^35^, we recently discovered that dyslipidemia may be an important upstream mediator of diastolic left ventricular dysfunction in the early stages of CKD^15^. LDL abnormality is a primary etiology of cardiovascular disease caused by dyslipidemia^36^. However, extensive lowering of plasma LDL does not prevent cardiovascular events in all patients. This prompted us to identify a culprit pathogenic LDL subfraction.

Our previous study has shown that increased LDL electronegativity in CKD disturbs intracellular calcium homeostasis resulting in cardiac diastolic dysfunction^15^. We have also found that electronegative LDL increases coronary artery disease risk in patients with uremia who are on maintenance hemodialysis^37^. In the current study, we demonstrate that the QT interval was prolonged in UNx rats when compared to the sham group. Incubation of normal cardiomyocytes with UNx LDL, which is more electronegative, reproduced a similar electrophysiological phenotype to that seen in UNx rat cardiomyocytes. Importantly, we have shown for the first time that electronegative LDL may underlie the electrophysiological remodeling via downregulation of KChIP2 protein expression in EPI cardiomyocytes with resultant parallel changes of the *I*_to_ current and altered channel kinetics in CKD. The disruption of the normal transmural gradient of *I*_to_ via downregulation of KChIP2 protein expression in combination with altered SERCA2a-regulated calcium homeostasis^15^ may further promote susceptibility to ventricular tachyarrhythmias in CKD.

In previous studies, we have shown that the electronegativity of LDL is increased in metabolic disorders and cardiovascular diseases, such as hypercholesterolemia, diabetes, metabolic syndrome, hypertension, myocardial infarction, and stroke^38^,^39^,^40^,^41^,^42^,^43^,^44^. However, in our UNx model, the rats did not show arterial hypertension, myocardial fibrosis, hyperglycemia, or gross structural heart disease^15^. Therefore, the increased LDL electronegativity in UNx-induced CKD may be a primary mediator of the electrophysiological remodeling rather than it being mediated secondarily through a common downstream effector pathway of general cardiovascular/metabolic disorders associated with CKD. Furthermore, the direct effects of small particles on ion channels usually occur within minutes of exposure. In the present study, none of the ion currents was altered within 10 minutes in UNx LDL-treated cardiomyocytes compared to controls; this finding indicates that it is unlikely that the effect of CKD-electronegative LDL on *I*_to_/APD is caused by a direct interaction of LDL on the ion channels, but rather it is caused by other indirect pathways. We have previously shown that the effect of electronegative LDL on vascular endothelial cells is mediated by the lectin-like oxidized LDL receptor (LOX-1). This could also be how electronegative LDL exerts its effects on cardiomyocytes, resulting in electrophysiological changes in CKD. The downstream signaling is still under investigation, but epigenetic regulation may be a possible mechanism^39^.

### Limitations

This study was undertaken to assess electrophysiological remodeling in early-stage CKD, which developed 8 weeks after UNx. We do not know if the changes in electrophysiological properties will remain constant or become adapted or maladapted in a more chronic scenario. However, the current UNx model is representative of an early, mild form of renal dysfunction; longer observation periods may show fibrosis and hypertrophy of the hearts, which may overtake the effects of early CKD on electrophysiological remodeling. Another limitation of our study is the significant difference in cardiac electrophysiology between rats and humans; rats have a higher heart rate, a more rapid repolarization, and a predominance of *I*_to_ over other repolarizing currents compared with humans. Further studies in larger mammal models are necessary to confirm our findings. Furthermore, because only limited amounts of LDL can be isolated from animals, we examined the effects of electronegative LDL on electrophysiological remodeling by incubating cardiomyocytes with UNx LDL instead of by directly injecting LDL into animals. Lastly, although we have identified a disruption in the normal transmural gradient of *I*_to_ in the current study and an altered intracellular calcium homeostasis in our previous study (which may sufficiently form a vulnerable ventricular substrate to generate ventricular tachyarrhythmias^10^,^15^,^32^), we did not perform a programmed electrical stimulation study to test the inducibility of ventricular tachyarrhythmias in UNx rats. Indeed, further studies are necessary to combine multiple modalities, including patch-clamp techniques, optical mapping, inserted loop recorders for arrhythmias detection, and programmed electrical stimulation, to investigate the electrophysiological substrate and susceptibility to ventricular arrhythmias in CKD.

## Conclusions

The results of our study indicate that CKD is associated with EPI-predominant prolongation of the APD and disruption of the physiological transmural gradient of *I*_to_ via downregulation of KChIP2 protein expression in the EPI region, which together may promote susceptibility to ventricular tachyarrhythmias. Electronegative LDL may underlie the downregulation of KChIP2 and the resultant electrophysiological remodeling in CKD.

## Materials and Methods

### Rat model of CKD

To characterize electrical changes in early CKD, we used a rat model of early CKD induced by UNx. All animal research was approved by the China Medical University Institutional Animal Care and Use Committee (2016-128), and all procedures were conducted in accordance with the Guide for the Care and Use of Laboratory Animals by the US National Institutes of Health. Adult male 8-week-old Sprague Dawley rats purchased from BioLASCO Taiwan Co., Ltd. (Taipei, Taiwan) were assigned to 1 of 2 groups: the UNx group or the sham group. All rats were anesthetized with 2% isoflurane (Abbott Laboratories, Abbott Park, IL, USA) and supported by a rodent ventilator (New England Medical Instruments, Medway, MA, USA). For UNx, the left kidney was removed immediately following ligation of the left renal artery and vein using a 4/0 silk suture. After 8 weeks, the biochemical parameters of blood samples including total cholesterol, triglyceride, LDL, HDL, BUN, and creatinine levels were measured by the Automated Biochemical Analyzer (SP-4430, Spotchem EZ, Arkray USA, Edina, MN, USA) with different strips.

### Electrocardiogram

The lead II surface electrocardiogram was recorded 8 weeks after the operation at the sampling rate of 1000 Hz by using PONEMAH real-time acquisition interface P3P Plus coupled to a digital converter (ML-870, ADInstruments, Colorado Springs, CO, USA). Data were analyzed by the software Lab Chart 7 plus (ADInstruments), and all characteristics were measured as the average of five consecutive cycles. The rate-corrected QTc interval was calculated according to the equation:


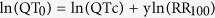


where QT_0_, QTc, y, RR100 are the observed QT, rate-corrected QT interval, value of the exponent, and normalized RR interval, respectively^17^.

### Isolation of cardiomyocytes

Left ventricular cardiomyocytes of rats were enzymatically isolated by the Langendorff perfusion method. Hearts were retrogradely perfused with Krebs buffer (in mmole/L): 120 NaCl, 12 glucose, 25 NaHCO_3_, 1.2 KH_2_PO_4_, 1.2 MgSO_4_, and 5.4 KCl; pH was adjusted to 7.4 by using HEPES. After a 5-min equilibration, we added 0.4 mg/mL collagenase (type II, Worthington) for 20 min. Hearts were then dissected from the left ventricular EPI and ENDO layer, leaving a distinct and dividing mid-myocardial layer. Single EPI or ENDO cardiomyocytes were then isolated by digestion with 0.4 mg/mL collagenase and 0.02 mg/mL trypsin (ThermoFisher Scientific, Waltham, MA, USA) in Krebs buffer for 20 min. After filtration, the cardiomyocytes were washed twice with Krebs buffer and stored in Krebs buffer. To examine the direct effect of LDL on control cardiomyocytes, we pretreated the cells with LDL isolated from sham and UNx rats for 12 hours.

### Electrophysiological recording

The whole-cell patch-clamp technique was used to record ionic currents and membrane potential with the Axon CNS 700B amplifier (Molecular Devices, LLC, Sunnyvale, CA, USA) with Digidata 1550 A data acquisition system and pClamp software (Version 10, Molecular Devices). A droplet of cell suspension was placed in a chamber mounted on the stage of an inverted microscope (Eclipse Ti-U, Nikon Corporation, Tokyo, Japan) in bath (extracellular) solution containing (in mM/L) 137 NaCl, 5.4 KCl, 1.8 CaCl_2_, 1.1 MgCl_2_, 6 HEPES, 22 glucose, and 0.33 NaH_2_PO_4_; pH was adjusted to 7.4 using NaOH at room temperature. Only quiescent rod-shaped cells showing clear-cross striations were studied. The mean capacitance of EPI cardiomyocytes from sham and UNx rats was 198.72 ± 20.57 and 192.79 ± 16.74 pF, respectively, and that of ENDO cardiomyocytes was 182.78 ± 12.38 and 186.89 ± 16.85, respectively (n = 20 per group). Heat-polished glass electrodes (tip resistances about 1.5 MΩ when filled with pipette internal solution) were prepared from borosilicate glass capillaries (outer diameter 1.5 mm) by the Glass Microelectrode Puller (PC-10, Narishige International Inc., East Meadow, NY, USA) and polished by the Microforge (MF-830, Narishige). The internal solution contained (in mmol/L) 120 KCl, 5 MgCl_2_, 5 MgATP, 10 HEPES, and 15 EGTA; the pH was adjusted to 7.2 using KOH at room temperature. Junctional potentials were zeroed before the formation of the membrane-pipette seal in the bath solution. Capacitances of cells were measured by calculating the total charge movement of the capacitative transient in response to a 10 mV hyperpolarizing pulse. *I*_to_ was defined as the difference between the peak value and the current level at the end of a 400 ms pulse. The cycle length of the stimulation in action potential measurements was 1000 ms. All patch-clamp experiments were performed at room temperature.

### LDL isolation and determination of LDL electronegativity

Rat plasma LDL was isolated by using sequential potassium bromide density-gradient ultracentrifugation (d = 1.030–1.063 g/mL). To confirm the quality and electronegativity of LDL samples, we separated them in 0.7% agarose by using electrophoresis and subjected the delipidated LDL samples to sodium dodecyl sulfate polyacrylamide gel electrophoresis.

### Immunoblotting

The left ventricular EPI layer of each UNx and sham rat was homogenized by using a Mini-Beadbeater-1 (BioSpec Products, Inc., Bartlesville, OK, USA) in T-PER Tissue Protein Extraction Reagent. For immunoblotting, polyclonal antibodies against Kv4.2/4.3 (Santa Cruz Biotechnology Inc., Santa Cruz, CA, USA), Kv1.4 (Academy Bio-Medical Company, Houston, TX, USA), KChIP2 (Merck Millipore, Darmstadt, Germany), and β-actin (Sigma Aldrich Corporation, St. Louis, MO, USA) were used.

### Statistical analysis

The data are expressed as the mean ± standard error of the mean, and the differences between the 2 groups were determined by using the Mann-Whitney U test. The difference between the mean EPI and ENDO *I*_to_ current was plotted as the transmural gradient of *I*_to_. The transmural gradient of *I*_to_ between sham and UNx rats was compared by using two-way ANOVA with the post-hoc Bonferroni correction. A *P*-value < 0.05 was considered statistically significant. The inactivation curve of *I*_to_ was fitted by using the Boltzmann equation:


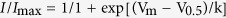


where *I* gives the current amplitude and *I*_max_ its maximum, V_m_ the potential of pulse, V_0.5_ the half-maximal inactivation potential, and k the slope factor. The recovery curve of *I*_to_ was fitted by the single exponential function:


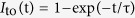


where τ is the time constant of decaying component of inactivation.

## Additional Information

**How to cite this article**: Lee, A.-S. *et al*. Electronegative LDL-Mediated Cardiac Electrical Remodeling in a Rat Model of Chronic Kidney Disease. *Sci. Rep.* **7**, 40676; doi: 10.1038/srep40676 (2017).

**Publisher's note:** Springer Nature remains neutral with regard to jurisdictional claims in published maps and institutional affiliations.

## Supplementary Material

Supplemental Figures

## Figures and Tables

**Table 1:** Biochemistry and electrocardiogram data for sham and unilateral nephrectomy (UNx) rats 8 weeks after operation.

	Sham (n = 6)	UNx (n = 6)	*P* value
**Biochemistry**			
Body weight (g)	368.33 ± 4.22	369.17 ± 10.83	0.94
Total cholesterol (mg/dL)	90.67 ± 4.39	91.83 ± 3.11	0.83
Triglyceride (mg/dL)	91.00 ± 4.98	86.83 ± 4.39	0.54
LDL (mg/dL)	32.13 ± 3.12	39.30 ± 3.78	0.17
HDL (mg/dL)	40.33 ± 2.51	35.17 ± 2.41	0.17
BUN (mg/dL)	15.33 ± 1.02	18.83 ± 1.01*	**0.04**
Creatinine (mg/dL)	0.28 ± 0.02	0.37 ± 0.02***	**<0.01**
**Electrocardiogram**			
RR interval (s)	0.20 ± 0.01	0.18 ± 2.66	0.59
Heart rate (bpm)	312.42 ± 24.73	332.98 ± 9.61	0.46
PR interval (s)	0.047 ± 0.003	0.046 ± 0.004	0.77
P duration (s)	0.024 ± 0.004	0.017 ± 0.001	0.13
QRS interval (s)	0.028 ± 0.002	0.032 ± 0.002	0.13
QT interval (s)	0.051 ± 0.005	0.082 ± 0.003**	<0.01
QTc (s)	0.037 ± 0.004	0.060 ± 0.002**	<0.01

Values are means ± S.E. LDL, low density lipoprotein; HDL, high density lipoprotein; BUN, blood urea nitrogen; QTc, corrected QT interval. *P < 0.05, **P < 0.01.
