# Supplementary material for: Electronegative LDL-mediated cardiac electrical remodeling in a rat model of chronic kidney disease
Source: Sci Rep. 2017 Jan 17;7:40676. doi: 10.1038/srep40676 (PMC5240592; doi:10.1038/srep40676)

Electronegative LDL-Mediated Cardiac Electrical Remodeling in a Rat Model of Chronic Kidney Disease

An-Sheng Lee, Wei-Yu Chen, Hua-Chen Chan, Ching-Hu Chung, Hsien-Yu Peng,
Chia-Ming Chang, Ming-Jai Su, Chu-Huang Chen, Kuan-Cheng Chang

Supplemental materials

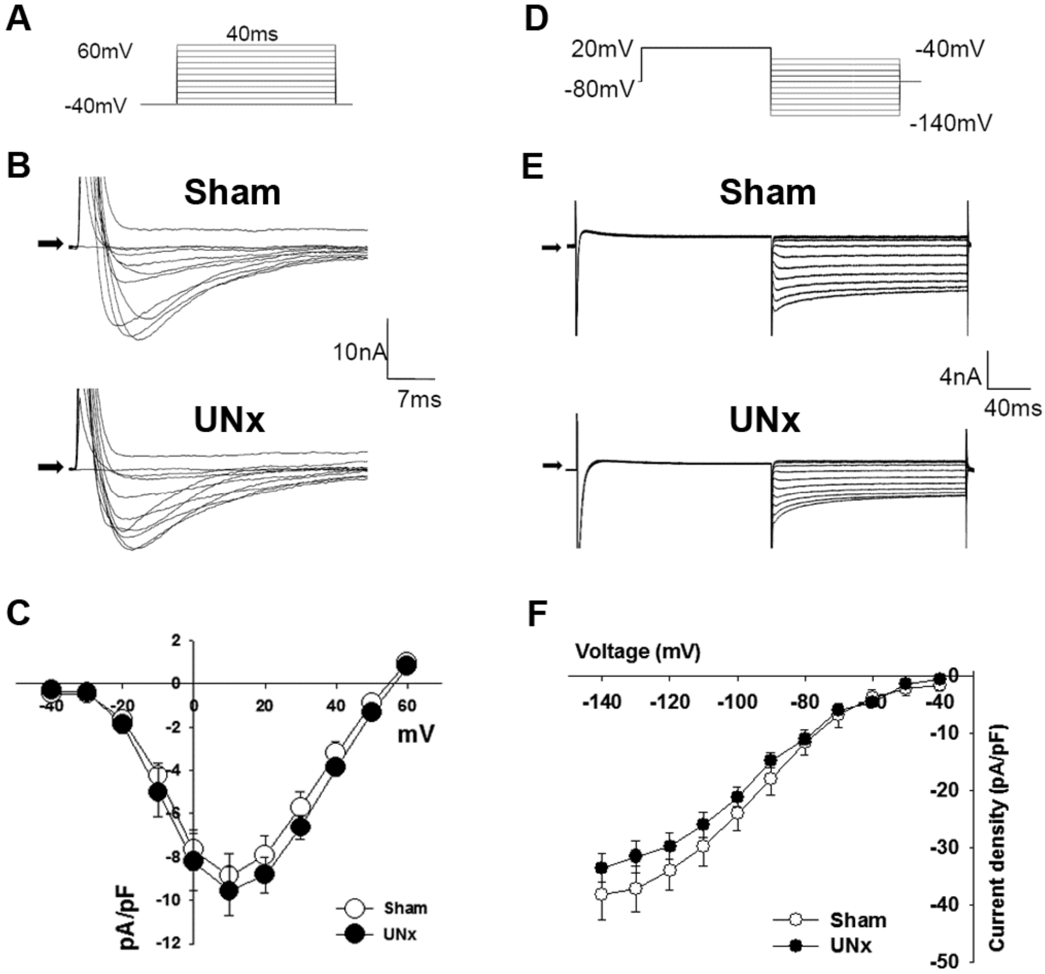

Supplemental Figure 1. There were no significant differences of the $I_{(Ca,L)}$ and I_{K1} currents between sham-operated cardiomyocytes and unilateral nephrectomy (UNx) cardiomyocytes. (A) Schematic diagram of the voltage clamp protocol for $I_{(Ca,L)}$ recordings. (B) The original superimposed records of $I_{(Ca,L)}$ are shown. The arrow head in each panel indicates the zero current level. (C) The I-V relationship for $I_{(Ca,L)}$ is shown (n=10 from 6 animals per group). (D) Schematic diagram of the voltage clamp protocol for I_{K1} recordings. (E) The original superimposed records of I_{K1} are shown. The arrow head in each panel indicates zero current level. (F) The I-V relationship of the current is shown (n=12 from 6 animals per group).

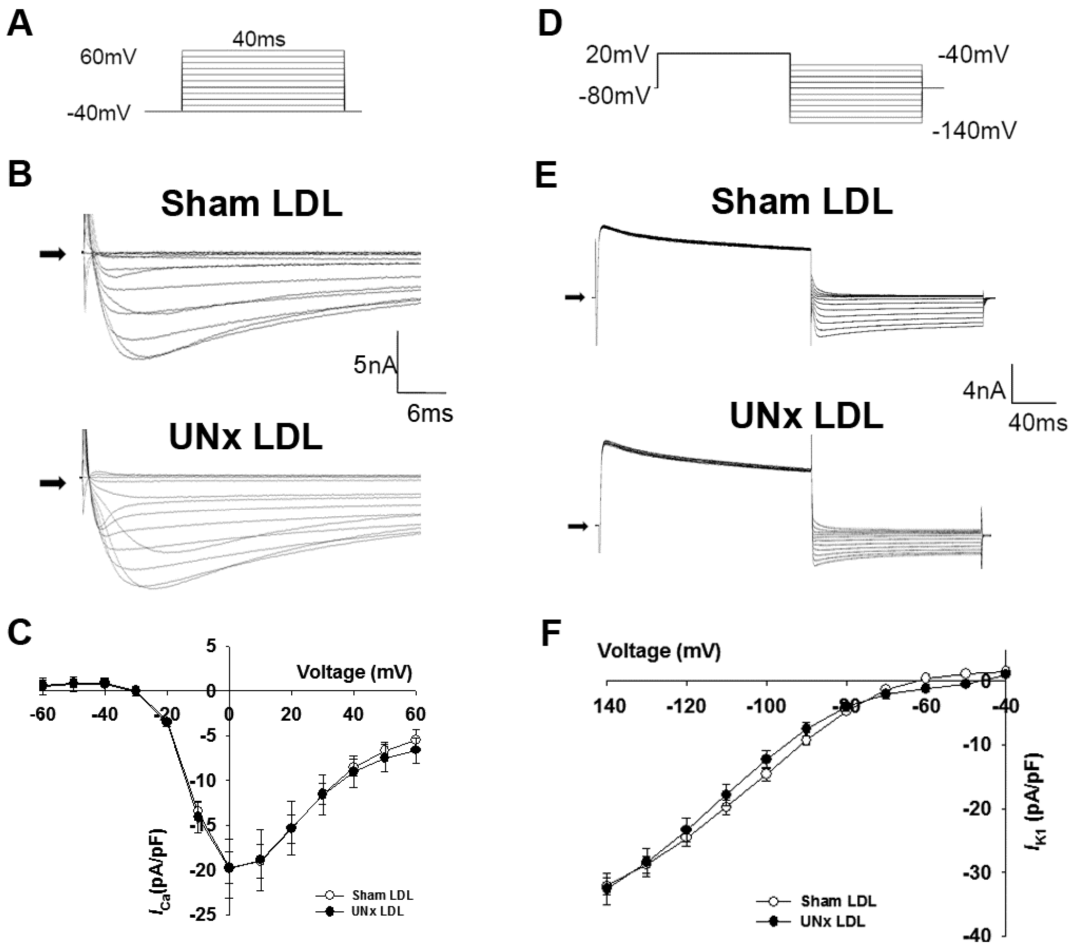

Supplemental Figure 2. There were no significant differences of the $I_{(Ca,L)}$ and I_{K1} currents between Sham LDL- and UNx LDL-treated normal cardiomyocytes. (A) Schematic diagram of the voltage clamp protocol for $I_{(Ca,L)}$ recordings. (B) The original superimposed records of $I_{(Ca,L)}$ are shown. The arrow head in each panel indicates zero current level. (C) The I-V relationship for $I_{(Ca,L)}$ is shown (n=7 from 4 animals per group). (D) Schematic diagram of the voltage clamp protocol for I_{K1} recordings. (E) The original superimposed records of I_{K1} are shown. The arrow head in each panel indicates zero current level. (F) The I-V relationships of current are shown (n=8 from 4 animals per group).

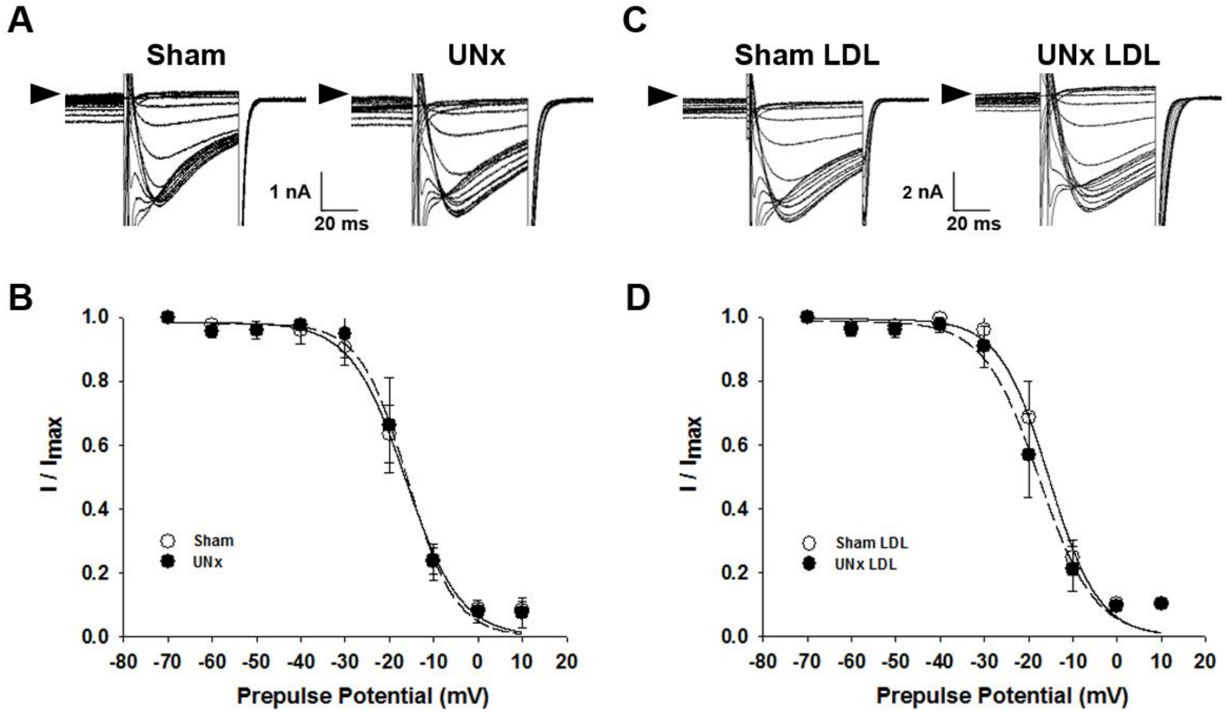

Supplemental Figure 3. There were no significant differences of the $I_{(Ca,L)}$ inactivation between sham-operated cardiomyocytes and unilateral nephrectomy (UNx) cardiomyocytes and between Sham LDL- and UNx LDL-treated normal cardiomyocytes. (A) The original superimposed records of the inactivation of $I_{(Ca,L)}$ are shown. The arrow head in each panel indicates zero current level. (B) The inactivation curve for $I_{(Ca,L)}$ is shown (n=6 from 4 animals per group). (C) The original superimposed records of the inactivation of $I_{(Ca,L)}$ are shown. The arrow head in each panel indicates zero current level. (D) The inactivation curve for $I_{(Ca,L)}$ is shown (n=7 from 4 animals per group).

Supplemental Figure 4.

KV4.3/4.2

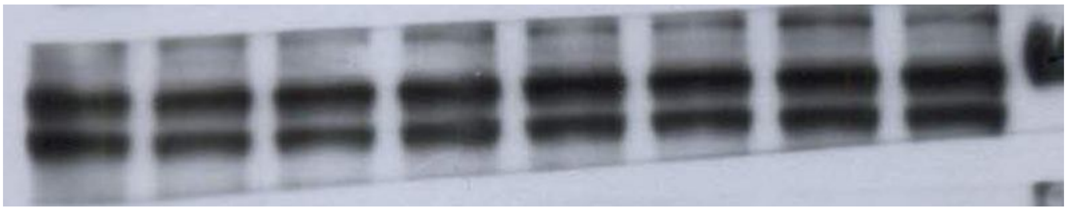

KV1.4

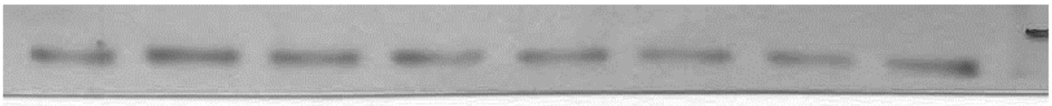

β-actin

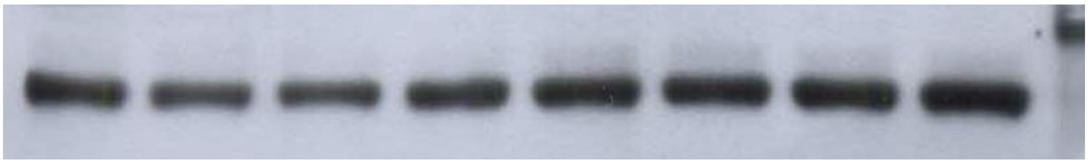

KChIP

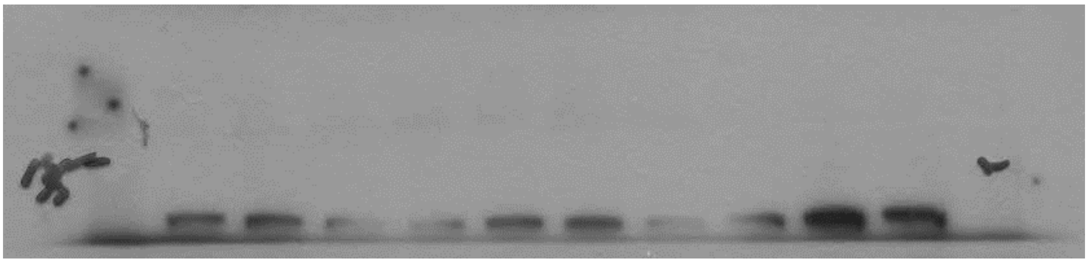

β-actin

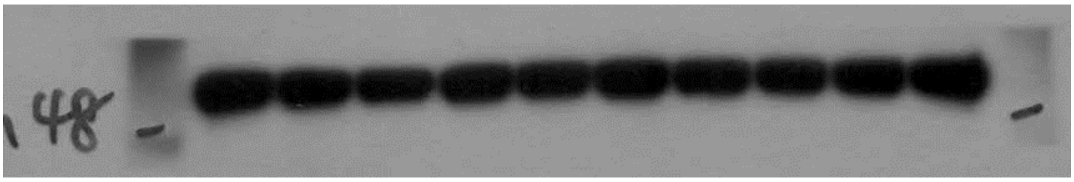

Supplement: Supplemental Figures [file srep40676-s1.pdf]
